# Supplementary material for: A chemical interpretation of protein electron density maps in the worldwide protein data bank
Source: PLoS One. 2020 Aug 12;15(8):e0236894. doi: 10.1371/journal.pone.0236894 (PMC7423092; doi:10.1371/journal.pone.0236894)
Supplement: S1 Table — (DOCX) [file pone.0236894.s001.docx]

**Table S1. Atom type mapping and the electron counts for the 20 common residues.**

| Residue | Atom types |
| --- | --- |
| GLY | N: N_single_bb, CA: C_single_bb, C: C_double_bb, O: O_double_bb, OXT: O_intermediate |
| ALA | N: N_single_bb, CA: C_single_bb, C: C_double_bb, O: O_double_bb, CB: C_single, OXT: O_intermediate |
| VAL | N: N_single_bb, CA: C_single_bb, C: C_double_bb, O: O_double_bb, CB: C_single, CG1: C_single, CG2: C_single, OXT: O_intermediate |
| LEU | N: N_single_bb, CA: C_single_bb, C: C_double_bb, O: O_double_bb, CB: C_single, CG: C_single, CD1: C_single, CD2: C_single, OXT: O_intermediate |
| ILE | N: N_single_bb, CA: C_single_bb, C: C_double_bb, O: O_double_bb, CB: C_single, CG1: C_single, CG2: C_single, CD1: C_single, OXT: O_intermediate |
| MET | N: N_single_bb, CA: C_single_bb, C: C_double_bb, O: O_double_bb, CB: C_single, CG: C_single, SD: S_single, CE: C_single, OXT: O_intermediate |
| PHE | N: N_single_bb, CA: C_single_bb, C: C_double_bb, O: O_double_bb, CB: C_single, CG: C_intermediate, CD1: C_intermediate, CD2: C_intermediate, CE1: C_intermediate, CE2: C_intermediate, CZ: C_intermediate, OXT: O_intermediate |
| TRP | N: N_single_bb, CA: C_single_bb, C: C_double_bb, O: O_double_bb, CB: C_single, CG: C_intermediate, CD1: C_intermediate, CD2: C_intermediate, NE1: N_intermediate, CE2: C_intermediate, CE3: C_intermediate, CZ2: C_intermediate, CZ3: C_intermediate, CH2: C_intermediate, OXT: O_intermediate |
| PRO | N: N_single_bb, CA: C_single_bb, C: C_double_bb, O: O_double_bb, CB: C_single, CG: C_single, CD: C_single, OXT: O_intermediate |
| SER | N: N_single_bb, CA: C_single_bb, C: C_double_bb, O: O_double_bb, CB: C_single, OG: O_single, OXT: O_intermediate |
| THR | N: N_single_bb, CA: C_single_bb, C: C_double_bb, O: O_double_bb, CB: C_single, OG1: O_single, CG2: C_single, OXT: O_intermediate |
| CYS | N: N_single_bb, CA: C_single_bb, C: C_double_bb, O: O_double_bb, CB: C_single, SG: S_single, OXT: O_intermediate |
| TYR | N: N_single_bb, CA: C_single_bb, C: C_double_bb, O: O_double_bb, CB: C_single, CG: C_intermediate, CD1: C_intermediate, CD2: C_intermediate, CE1: C_intermediate, CE2: C_intermediate, CZ: C_intermediate, OH: O_single, OXT: O_intermediate |
| ASN | N: N_single_bb, CA: C_single_bb, C: C_double_bb, O: O_double_bb, CB: C_single, CG: C_double, OD1: O_double, ND2: N_single, OXT: O_intermediate, |
| GLN | N: N_single_bb, CA: C_single_bb, C: C_double_bb, O: O_double_bb, CB: C_single, CG: C_single, CD: C_double, OE1: O_double, NE2: N_single, OXT: O_intermediate |
| ASP | N: N_single_bb, CA: C_single_bb, C: C_double_bb, O: O_double_bb, CB: C_single, CG: C_double, OD1: O_intermediate, OD2: O_intermediate, OXT: O_intermediate |
| GLU | N: N_single_bb, CA: C_single_bb, C: C_double_bb, O: O_double_bb, CB: C_single, CG: C_single, CD: C_double, OE1: O_intermediate, OE2: O_intermediate, OXT: O_intermediate |
| LYS | N: N_single_bb, CA: C_single_bb, C: C_double_bb, O: O_double_bb, CB: C_single, CG: C_single, CD: C_single, CE: C_single, NZ: N_single, OXT: O_intermediate |
| ARG | N: N_single_bb, CA: C_single_bb, C: C_double_bb, O: O_double_bb, CB: C_single, CG: C_single, CD: C_single, NE: N_intermediate, CZ: C_double, NH1: N_intermediate, NH2: N_intermediate, OXT: O_intermediate |
| HIS | N: N_single_bb, CA: C_single_bb, C: C_double_bb, O: O_double_bb, CB: C_single, CG: C_intermediate, ND1: N_intermediate, CD2: C_intermediate, CE1: C_intermediate, NE2: N_intermediate, OXT: O_intermediate |
