# Supplementary material for: A chemical interpretation of protein electron density maps in the worldwide protein data bank
Source: PLoS One. 2020 Aug 12;15(8):e0236894. doi: 10.1371/journal.pone.0236894 (PMC7423092; doi:10.1371/journal.pone.0236894)
Supplement: S2 Table — (DOCX) [file pone.0236894.s002.docx]

**Table S2. Atom-specific electron counts for the 20 common residues.**

| Residue | Number of electrons |
| --- | --- |
| GLY | N: 8, CA: 8, C: 6, O: 8, OXT: 8 |
| ALA | N: 8, CA: 7, C: 6, O: 8, CB: 9, OXT: 8 |
| VAL | N: 8, CA: 7, C: 6, O: 8, CB: 7, CG1: 9, CG2: 9, OXT: 8 |
| LEU | N: 8, CA: 7, C: 6, O: 8, CB: 8, CG: 7, CD1: 9, CD2: 9, OXT: 8 |
| ILE | N: 8, CA: 7, C: 6, O: 8, CB: 7, CG1: 8, CG2: 9, CD1: 9, OXT: 8 |
| MET | N: 8, CA: 7, C: 6, O: 8, CB: 8, CG: 8, SD: 16, CE: 9, OXT: 8 |
| PHE | N: 8, CA: 7, C: 6, O: 8, CB: 8, CG: 6, CD1: 7, CD2: 7, CE1: 7, CE2: 7, CZ: 7, OXT: 8 |
| TRP | N: 8, CA: 7, C: 6, O: 8, CB: 8, CG: 6, CD1: 7, CD2: 6, NE1: 8, CE2: 6, CE3: 7, CZ2: 7, CZ3: 7, CH2: 7, OXT: 8 |
| PRO | N: 7, CA: 7, C: 6, O: 8, CB: 8, CG: 8, CD: 8, OXT: 8 |
| SER | N: 8, CA: 7, C: 6, O: 8, CB: 8, OG: 9, OXT: 8 |
| THR | N: 8, CA: 7, C: 6, O: 8, CB: 7, OG1: 9, CG2: 9, OXT: 8 |
| CYS | N: 8, CA: 7, C: 6, O: 8, CB: 8, SG: 17, OXT: 8 |
| TYR | N: 8, CA: 7, C: 6, O: 8, CB: 8, CG: 6, CD1: 7, CD2: 7, CE1: 7, CE2: 7, CZ: 6, OH: 9, OXT: 8 |
| ASN | N: 8, CA: 7, C: 6, O: 8, CB: 8, CG: 6, OD1: 8, ND2: 9, OXT: 8 |
| GLN | N: 8, CA: 7, C: 6, O: 8, CB: 8, CG: 8, CD: 6, OE1: 8, NE2: 9, OXT: 8 |
| ASP | N: 8, CA: 7, C: 6, O: 8, CB: 8, CG: 6, OD1: 8, OD2: 8, OXT: 8 |
| GLU | N: 8, CA: 7, C: 6, O: 8, CB: 8, CG: 8, CD: 6, OE1: 8, OE2: 8, OXT: 8 |
| LYS | N: 8, CA: 7, C: 6, O: 8, CB: 8, CG: 8, CD: 8, CE: 8, NZ: 9, OXT: 8 |
| ARG | N: 8, CA: 7, C: 6, O: 8, CB: 8, CG: 8, CD: 8, NE: 8, CZ: 6, NH1: 8, NH2: 8, OXT: 8 |
| HIS | N: 8, CA: 7, C: 6, O: 8, CB: 8, CG: 6, ND1: 7, CD2: 7, CE1: 7, NE2: 8, OXT: 8 |
