# Supplementary figures and images for: A chemical interpretation of protein electron density maps in the worldwide protein data bank
Source: PLoS One. 2020 Aug 12;15(8):e0236894. doi: 10.1371/journal.pone.0236894 (PMC7423092; doi:10.1371/journal.pone.0236894)

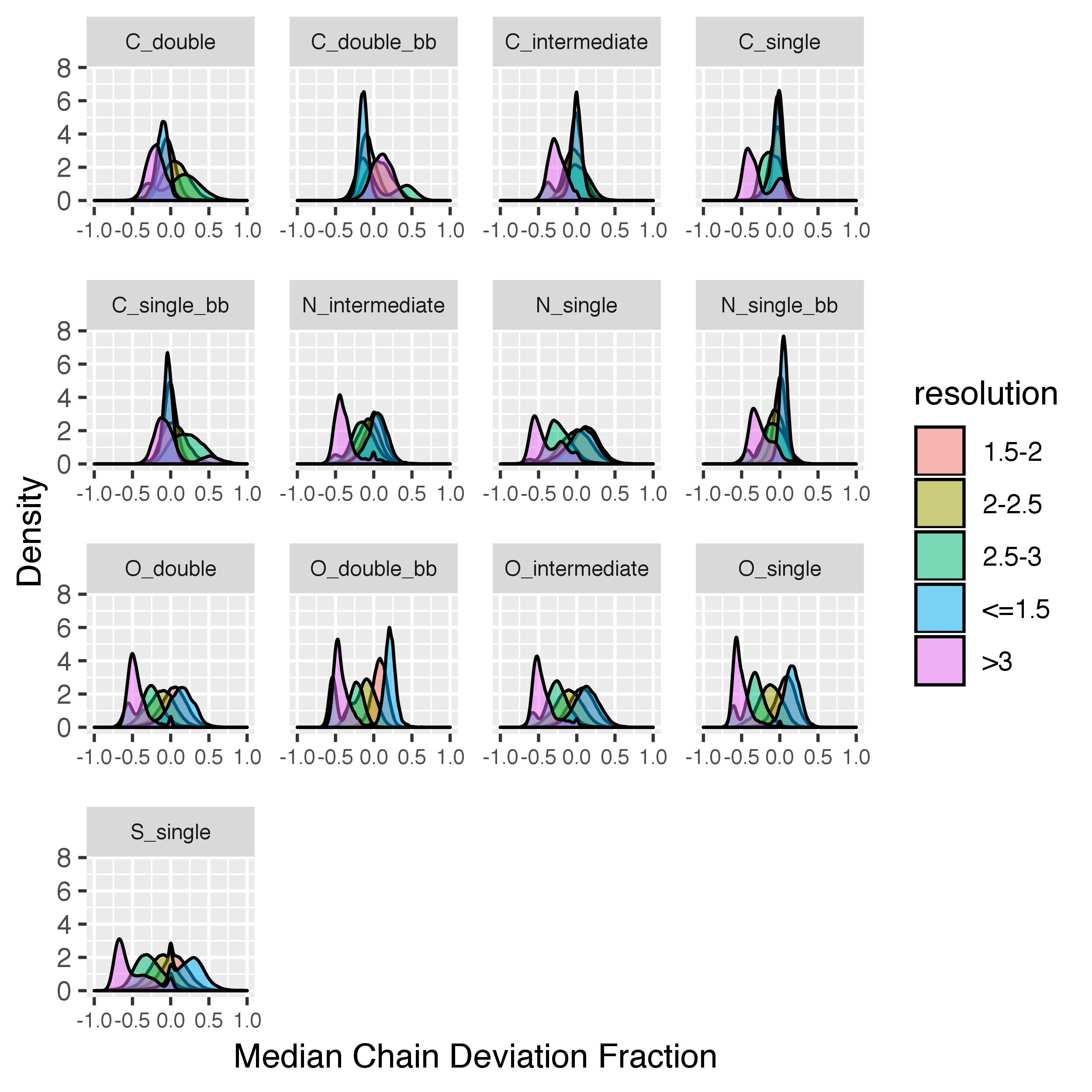

Supplement: S1 Fig — (TIFF) [file pone.0236894.s003.tiff]
