# Supplementary material for: A chemical interpretation of protein electron density maps in the worldwide protein data bank
Source: PLoS One. 2020 Aug 12;15(8):e0236894. doi: 10.1371/journal.pone.0236894 (PMC7423092; doi:10.1371/journal.pone.0236894)
Supplement: Attachment — Submitted filename: PLOS-1-20-10145.pdf [file pone.0236894.s004.pdf]

Review of PLOS ONE ms PONE-D-20-10145 "A chemical interpretation of protein electron density maps in the worldwide protein data bank" by S.Yao & H.N.B.Moseley

The goal of this work is very commendable (to generate electron density on absolute level) and the solution is elaborated through a convoluted algorithm, which - although basically, I think, correct and well documented - is… unnecessary. In principle, the Authors arrive at the "arbitrary" => "absolute" scale factor by a tedious comparison of the experimental electron density map with its atomic model. Or in other words, they want to scale apples with oranges. But a much simpler solution exists: compare oranges with oranges. In the simpler approach one would first convert the atomic model to its Fourier Transform Fc(hkl), and then use a subset of those calculated structure factors corresponding exactly to the set of experimental Fo(hkl) data, to generate ρc(xyz), i.e. the calculated (Fc) electron density map. This map would be the target for scaling the experimental (observed) ρo(xyz) electron density map (Fo). The scaling would be of course linear: ρc(xyz) = $a \cdot \rho o(xyz) + b$, and would have to be fulfilled at all grid points on which the two maps have been calculated. Since this strictly linear problem is hugely overdetermined, only the most reliable grid points could be included, e.g. within 1 A of the atomic centers of the model, or indeed within the atomic radii used in the ms. The set of linear equations could be solved by the method of least squares, with the addition of a robust method to filter out outliers, e.g. if some fragments of the model are of poor quality. This way the best values of the *a* and *b* parameters are obtained. I have not tried this algorithm myself - it's not my paper. But I am pretty sure it should work quite well. At least the Authors should try a simple method first, before proposing an algorithm that may be unduly complicated.

In conclusion, I cannot recommend acceptance of this paper in its present form. In addition to the doubts outlined above, there is one more misgiving. Assuming that we have electron density re-scaled to absolute units by one method or the other, the real question is : "So what?" The Authors should provide examples to clearly demonstrate what is possible with their maps that would not be possible with sigma-scaled 2Fo-Fc and Fo-Fc maps. Right now there is a lot of verbal promise but very little of concrete proof. BTW, the caption of Fig. 10 is amazingly unhelpful.

It may be irrelevant in view of my final recommendation (reject), but since I've read the paper very carefully, I'm also including my more specific comments and critical remarks, divided into "Substantive" and "Technical".

Substantive problems

The convoluted algorithm includes a series of corrections, one after another. At some point the reader is lost as to the purpose of all those corrections. Perhaps it would be helpful to add a graph showing the distribution of the sample 1000 structures?

The Authors should calibrate their method with ultrahigh-resolution PDB crystal structures which provide accurate estimate of electron density levels in e/A^3 because they have a large Ewald sphere of very accurate F(hkl) data and in addition allow reliable estimation of F(000) since the (nearly) complete atomic content of the unit cell is practically known. The recommended examples would be the PDB entries 3NIR (0.48 A, crambin, highest resolution but unsatisfactory refinement), 1EJG (0.54 A, crambin) (comparison of the two crambin structures would provide an interesting "internal standard") and 2VB1 (0.65 A, lysozyme) for proteins, and 3P4J (0.55 A, Z-DNA) for nucleic acids.

I also note that, infrequent as they are, occasionally ultrahigh-resolution macromolecular structures are presented with the electron density maps expressed on the absolute scale (e.g. Addlagatta et al. (2001) Acta Cryst. D57: 649-663). Such maps could also be used to validate the scaling procedures proposed in this work.

p3, “These low-quality regions arise from structural model and electron density mismatches…”, actually, most often the low-quality regions arise because of **absence** of electron density.

p6, I am surprised that the Authors have completely overlooked the paper by Tickle (2012) Acta Cryst. D68: 454-467, which is the standard classical reference for sigma-contoured electron density maps and more. Also, there is a good discussion there about the radii of atoms that cover 95% of electron density, and about the influence of B-factors and resolution (see 5.6. The limiting radius of the atomic density; as well as Table 3 and Fig. 11 therein).

Fig. 2 caption; it is correct to include H atoms in the electronic inventory of their “carriers” if H atoms were not included in Fc calculations. However, as is very often the case, if H atoms were included in Fc, then this strategy is incorrect. Moreover, if the electron density of H atoms is added to the bound atom, then interpretation by a simple spherical volume around that atom will lead to systematic errors. In addition, the resolution of the electron density map is also important.

I don’t understand the idea of dividing the formula for F(000) in eq. (6) by V. Structure factors F (including F(000), of course), are expressed directly in electrons (e). Also, counting the electron contribution of the solvent molecules is necessary, but in most cases we miss a lot of water molecules (not included in the model). At low resolution, we cannot **count** the water molecules at all. What could be done, however, would be to estimate the number of water molecules from Matthews volume and specific density of water (1 g/cm^3) and add their electrons to F(000).

I wonder, how much the re-scaled difference maps show-cased on p15 and Fig. 10 would differ from normal mFo-DFc and 2mFo-DFc maps. I think such a comparison should be illustrated.

The method should be extended (in the future?) to explicitly apply to nucleic acids as well. Nucleic acids constitute an important segment of PDB structures.

To be of general applicability, a method like this should produce electron density maps in mtz or other ccp4/coot/pymol-readable format, so that they could be easily loaded and displayed for visualization.

Technical remarks

p3, “activities happens”, singl./pl. problem.

p6 and elsewhere, the Greek letter sigma (and other symbols) is misprinted as a funny character.

I couldn’t find anywhere in the ms the key information about the density of the grid over which the electron density is calculated and analyzed.

Fig. 2 caption, “20 common amino acid”, singl./pl. problem.

Correct the grammar in Fig. 2 (“by atom the b-factor”).

p7, “total number of electrons are”, singl./pl. problem.

On p8, the term “chain deviation fraction” is suddenly changed to “chain fraction” (without definition).

p8 and elsewhere, “on 100 random structures”, change to “of…”.

p10, “Fig. 3. Sina plots of density ratio for atoms, residues, and chains”, perhaps it would be helpful to add a short description a sina plots, for example as in https://ggforce.data-imaginist.com/reference/geom_sina.html: “Sina plot is an enhanced jitter strip chart, where the width of the jitter is controlled by the density distribution of the data within each class.”.

Fig. 3, frankly, I don’t see much difference between the top and bottom panels… Moreover, it seems to me that the x axis of panels C and F shows residues, not chain IDs; I am confused…

The “optimized” atomic radii are usually ≥ the “original” radii. An outstanding exception in Table 1 is S. Any reason why?

p13, last sentence before new section, don’t begin sentence with “And…”.

p13, explain “distribution modes”.

p13, “It was then applied to the all PDB structures that has usable electron density data”, several grammatical errors in this sentence.

Fig. 8 caption talks about 2mFo-DFc maps, but panels A/B are supposed to correspond to 2Fo-Fc/Fo-Fc maps.

Something wrong with the first sentence of Fig. 9 caption.

p14, “with a local region.”, perhaps “within a local region.”.

p15, difference electron density of “16 and 29 e” sounds like serious overshooting. Should be metal or halide ions, or at least S.

Fig. S1, the axes need proper labels and units. The values of the ticks on the x axes are squeezed too much and unreadable. The plots, with the current caption, are not very useful.

Tables S1 and S2 with the inventory of bonds and atoms in standard amino acid residues are banal and could be omitted.

Please note that the proper symbol to be used for Atomic Displacement Parameter (temperature factor) is B-factor.

I am glad to note that the GITHUB documentation and examples seem to work well. I note that GITHUB reports version 1.0.1. and PythonPackageIndex version 1.1.0. There are also some shortcomings and bugs. For example, the command:

python3 -m pdb_eda single 3han 3han.all.csv --all --out-format=csv

instead of human-readable printouts, returns a printout of rather useless python objects.

Execution of:

python3 -m pdb_eda single 3han 3han.all.csv --all --out-format=csv

generates an error message:

AttributeError: 'int' object has no attribute 'aggregateCloud'

which is not very helpful.
