# Supplementary material for: A chemical interpretation of protein electron density maps in the worldwide protein data bank
Source: PLoS One. 2020 Aug 12;15(8):e0236894. doi: 10.1371/journal.pone.0236894 (PMC7423092; doi:10.1371/journal.pone.0236894)
Supplement: Attachment — Submitted filename: response_to_reviewer_comments_revision1_v1.docx [file pone.0236894.s006.docx]

**Reviewer 3:**

*In view of the rebuttal, in particular emphasising that the audience of this paper are not professional crystallographers but less trained consumers of the PDB, as well as highlighting that the application of the proposed method and software lies in PDB-wide analyses, I reluctantly tend to agree that this manuscript could be accepted after additional minor revision. I am also amused by the correct unmasking of Referee 3 (i.e. myself) as a (well trained, I supposed) crystallographer! However, I have the impression that in many cases the Authors have glossed over the highlighted problems too easily, sometimes actually evading to address the issue at hand directly.*

**Response:**

We thank the reviewer for seeing the utility of our software for the general consumer of the PDB. We were not trying to gloss over issues raised by the reviewers, but there were quite a few issues raised by all three reviewers, some issues with more merit than others. The revisions and response document take real effort to generate.

**Issue 1:**

*One such remark is “my” Issue 6 (first instance with this issue number, as the Authors’ numbering of issues has errors), in which I pointed out that F(000) (as any other structure factor) is expressed in electrons. Therefore, the division of formula (6) by unit cell volume (V) is incorrect.*

**Response:**

We misunderstood the point being made here the first time, because we were focused on how to use F(000) for correction. Thank you for raising it again! We would not want this kind of definition error in the published literature. We have made the following revisions:

“Unfortunately, not all structure factor programs provide the F_000_ value. So as an estimation, we add up the numbers of electrons for all the atoms of a model in the unit cell, including symmetry structure units and modeled water molecules,

F_000_ = $\sum n_{t}Z_{t}$	(6)

Where n_t_ is number of atoms of element t in the asymmetric unit and Z_t_ is the number of electrons (atomic number) of element t. This estimated F_000_ term is then divided by the unit cell volume V in Å^3^ and added to the density values of all the voxels.

”

**Issue 2:**

*Also, I think that the Authors are too quick to dismiss bug reports and to defer action to the future. Reports like the one below are useless for untrained users and can be certainly handled in a better way by error-handling routines included in the software package.*

*>> python3 -m pdb_eda single 6xyz 6xyz.atom.csv --atom --out-format=csv*

*>> AttributeError: 'int' object has no attribute 'aggregateCloud'*

**Response:**

It is not that we are dismissing a bug report. We handle all issues posted on our GitHub repositories. Please look at <https://github.com/MoseleyBioinformaticsLab> to see where we address issues raised by users (for example, <https://github.com/MoseleyBioinformaticsLab/GOcats/issues>). However, we cannot anticipate all possible errors raised by the Python interpreter and provide a meaningful response to the user. All we can reasonably do is fix bugs and return more meaningful error messages for the bugs or edge cases that we find ourselves or are pointed out to us by others. Towards this end, we have verified the error and concluded that it is due to a flaw in the command line interface where object creation was not correctly tested for success. We have created an issue on our GitHub pdb-eda repository based on what the reviewer has provided and our testing of the error: <https://github.com/MoseleyBioinformaticsLab/pdb_eda/issues/1> . We expect to have this issue fixed when we roll out a larger set of updates to the codebase in the next 2-4 weeks. Please be patient with us.
